# Advocating for efforts to protect African children, families, and communities from the threat of infectious diseases: report of the First International African Vaccinology Conference

**DOI:** 10.11604/pamj.2016.23.53.9097

**Published:** 2016-02-29

**Authors:** Charles Shey Wiysonge, Zainab Waggie, Anthony Hawkridge, Barry Schoub, Shabir Ahmed Madhi, Helen Rees, Gregory Hussey

**Affiliations:** 1 Vaccines for Africa Initiative, Institute of Infectious Disease and Molecular Medicine, Faculty of Health Sciences, University of Cape Town, Cape Town, South Africa; 2 Centre for Evidence-based Health Care, Faculty of Medicine and Health Sciences, Stellenbosch University, Cape Town, South Africa; 3 Division of Medical Microbiology, Faculty of Health Sciences, University of Cape Town, Cape Town, South Africa; 4 Department of Health, Provincial Government of the Western Cape, Cape Town, South Africa; 5 National Institute for Communicable Diseases, National Health Laboratory Service, Johannesburg, South Africa; 6 Medical Research Council: Respiratory and Meningeal Pathogens Research Unit, University of the Witwatersrand, Johannesburg, South Africa; 7 Wits Reproductive Health and HIV Institute, University of the Witwatersrand, Johannesburg, South Africa

**Keywords:** Africa, immunisation, vaccine-preventable diseases, vaccine advocacy, GVAP

## Abstract

One means of improving healthcare workers’ knowledge of and attitudes to vaccines is through running vaccine conferences which are accessible, affordable, and relevant to their everyday work. Various vaccinology conferences are held each year worldwide. These meetings focus heavily on basic science with much discussion about new developments in vaccines, and relatively little coverage of policy, advocacy, and communication issues. A negligible proportion of delegates at these conferences come from Africa, home to almost 40% of the global burden of vaccine-preventable diseases. To the best of our knowledge, no major vaccinology conference has ever been held on the African continent apart from World Health Organization (WHO) meetings. The content of the first International African Vaccinology Conference was planned to be different; to focus on the science, with a major part of discussions being on clinical, programmatic, policy, and advocacy issues. The conference was held in Cape Town, South Africa, from 8 to 11 November 2012. The theme of the conference was “Advocating for efforts to protect African children, families, and communities from the threat of infectious diseases”. There were more than 550 registered participants from 55 countries (including 37 African countries). There were nine pre-conference workshops, ten plenary sessions, and 150 oral and poster presentations. The conference discussed the challenges to universal immunisation in Africa as well as the promotion of dialogue and communication on immunisation among all stakeholders. There was general acknowledgment that giant strides have been made in Africa since the global launch of the Expanded Programme on Immunisation in 1974. For example, there has been significant progress in introducing new and under-utilised vaccines; including hepatitis B, Haemophilus influenza type b, pneumococcal conjugate, rotavirus, meningococcal A conjugate, and human papillomavirus vaccines. In May 2012, African countries endorsed the Global Vaccine Action Plan at the World Health Assembly. However, more than six million children remain incompletely vaccinated in Africa leading to more than one million vaccine-preventable deaths annually. In addition, there are persistent problems with leadership and planning, vaccine stock management, supply chain capacity and quality, provider-parent communication, and financial sustainability. The conference delegates agreed to move from talking to taking concrete actions around children's health, and to ensure that African governments commit to saving children's lives. They would advocate for lower costs of immunisation programmes in Africa, perhaps through bulk buying and improved administration of vaccine rollout through the New Partnership for Africa's Development.

## Introduction

In May 2012 African countries joined other member states of the World Health Assembly to endorse the Global Vaccine Action Plan (GVAP), an agenda to avert millions of vaccine-preventable deaths by 2020 through impartial access to existing vaccines for people in every community around the world [1]. At the time of endorsing the GVAP, despite substantial improvements with immunisation programmes in Africa, there were persistent problems with leadership and planning, vaccine stock management, supply chain capacity and quality, provider-parent communication, and financial sustainability [2]. Childhood immunisation coverage varied widely between and within African countries, and more than seven million children on the continent did not receive the full series of vaccines in their national immunisation schedule [3]. The consequences of low immunisation coverage include the premature deaths of more than one million children each year in Africa [4]. Many reasons have been proposed as to why vaccination coverage in African children is low [3, 5, 6]. These include inconsistent policies, poor understanding of diseases, poverty, and cultural issues. Other factors which have been blamed include parental apathy or ignorance and high cost of vaccines. In addition, the fragmented nature of healthcare systems in some countries, with children′s records scattered among many providers they might see during the time they were supposed to receive their vaccines, may lead to missed opportunities to vaccinate. Having a forum such as a vaccinology conference with delegates from all African countries, will allow dialogue to identify the biggest challenges faced and opportunities to identify solutions employed by other countries. One means of improving healthcare workers’ knowledge of and attitudes to existing vaccines is through running vaccinology conferences which are accessible, affordable and relevant to their everyday work. Each year various international vaccinology conferences are organised around the world (http://www.isv-online.org/). However, the focus of these meetings is heavily on basic science, with much discussion about new developments in vaccines, but relatively little coverage of policy, advocacy, and communication issues. The meetings attract delegates mainly from Europe and North America. For example, at the 2011 International Society for Vaccines Annual Congress (http://isvcongress.org/programme/5th-isv-annual-congress-program), less than 3% of participants were from Africa (home to over 15% of the world's population and where almost 40% of the burden of vaccine preventable diseases occurs). To the best of our knowledge, no major vaccinology conference has ever been held on the African continent apart from World Health Organization (WHO) meetings. We therefore proposed the International African Vaccinology Conference, whose content would be different from the others. While the meeting would focus on the science a major part of the discussions would be on clinical, programmatic, policy, and advocacy issues.

## Conference outlines and outcomes

The first International African Vaccinology Conference took place in Cape Town, South Africa, from 8 to 11 November 2012. The objectives of the conference were to discuss challenges and obstacles to achieving universal childhood immunisation coverage in Africa, to identify and open dialogue with all immunisation stakeholders on the continent, and to promote communication between countries in Africa. The conference was organised by the Vaccines for Africa Initiative based at the University of Cape Town, and the National Institute for Communicable Diseases, a branch of the (South African) National Health Laboratory Service. It brought together healthcare workers; immunisation programme staff; researchers; civil society (including Médecins Sans Frontières); United Nations’ agencies (World Health Organization, United Nations Children's Fund); international organisations(Malaria Vaccine Initiative, PATH, International AIDS Vaccine Initiative, Agence de Medecine Preventive, Bill and Melinda Gates Foundation); private-public partnerships (Global Alliance for Vaccines and Immunization, Biovac Institute, Polio Research Foundation); industry (GlaxoSmithKline, Sanofi Pasteur, MSD, Pfizer, and Emergent Biosolutions); product development partners (Aeras Global TB Vaccine Initiative); national regulatory agencies; and government agencies (Centers for Disease Control and Prevention, National Institutes of Health). The conference was held at the Lagoon Beach Hotel and Conference in Cape Town, South Africa. There were 572 registered participants from 37 African countries as well as delegates from countries in the Americas, Asia, Australia, and Europe. The official language of the conference was English, with simultaneous translation to French. The conference was preceded by nine pre-conference workshops. This included society meetings: the Foundation launch of the African Paediatric Infectious Diseases Society (AfPIDS), the 7^th^ African Rotavirus Symposium, and the Influenza in Africa workshop [7]. Other workshops addressed essential vaccine issues in Africa: surveillance of adverse events following immunisation; communication, advocacy and social mobilisation; role of partially effective vaccines; evidence-based approaches to immunisation policies; and national immunisation technical advisory groups (NITAGs). The conference also hosted industry-sponsored workshops which covered a wide range of topics, including: pneumococcal, human papilloma virus (HPV), dengue, influenza, rotavirus, and tuberculosis (TB) vaccines. Emerging African researchers were offered an opportunity to show case their work. One hundred and thirty four abstracts were submitted by emerging African researchers and 118 were selected for presentation: 37 oral papers, and 81 poster presentations. They covered a comprehensive set of topics relevant to immunisation programmes in Africa, including: ethics, operational issues, surveillance, existing vaccines, and the vaccine pipeline (particularly for TB and malaria vaccines). There were ten plenary sessions, namely: progress towards reaching the Millennium Development Goals (MDGs), vaccine success stories, challenges in controlling infections, vaccine economics and financing, vaccine development and production in sub-Saharan Africa, critical operational issues, Decade of Vaccines / Global Vaccine Action Plan (GVAP), new vaccines, access, and closure. There were 36 plenary speakers, 20 of whom hailed from Africa. Launching the conference, the Dean of the Faculty of Health Sciences at the University of Cape Town in South Africa, Professor Marian Jacobs said lack of access to immunisation is a denial of children's basic human rights. “The title International African Vaccinology Conference captures the spirit and intent of the gathering - to signal the urgent need to secure a global commitment to addressing the challenges to achieving universal childhood immunisation in Africa, she continued. All African countries (except Somalia) have ratified the Convention on the Rights of a Child, which reminds us of the legal entitlement of every African child to the right to survival, to universal access to health care, and especially to universal access to preventive measures such as immunisation. “So your vaccine efforts … have to be underpinned by this value if we are to address the inequity which plagues immunisation access”, she concluded.

There was general acknowledgment that giant strides have been made since the global launch of the Expanded Programme on Immunisation in 1974. It is now estimated that about 2.5 million children are saved every year due to vaccines against infectious diseases. There has been substantial support and funding for low-income African countries through mechanisms like the Global Alliance for Vaccines and Immunisation (GAVI), enabling countries to introduce new life-saving vaccines; but progress has been slow. Vaccine development has also advanced, with new vaccines being brought to the market and others in late stages of clinical trials. The time from registration of a new vaccine to introduction in low-income countries has been significantly reduced, with the introduction of new pneumococcal and rotavirus vaccines in low-income countries (with GAVI and industry support) occurring almost at the same time as in high-income countries. These vaccines prevent the leading causes of child mortality, namely: pneumonia and diarrhoea. We also have the new meningococcal A conjugate vaccine manufactured solely for use in the African meningitis belt. In December 2010, global health leaders committed to making the next 10 years the Decade of Vaccines - to ensure discovery, development, and delivery of life-saving vaccines globally, especially to the poorest countries. This culminated with the endorsement in May 2012 of the Global Vaccine Action Plan. In spite of all these initiatives, more than six million children remain incompletely vaccinated in Africa leading to more than one million deaths annually. This sub-optimal coverage had a huge impact on the inability of Nigeria to interrupt wild poliovirus transmission (until 2014), with importation to other African countries. The lessons learnt from India (declared polio free on 25 February 2012), in interrupting wild poliovirus transmission, were presented. The main factors that delayed the interruption of transmission were an extremely high force of transmission, failure to vaccinate, and vaccine failure. The issue of low coverage and missed opportunities for vaccination was addressed by eight to nine campaigns annually for children under the age of five years, micro-planning of the campaigns to cover every household, and catching up with migrant children in transit. The failure of the trivalent oral poliovirus vaccine to induce adequate vaccine efficacy was addressed by the manufacture, testing, and introduction of mono valent and bivalent oral poliovirus vaccines. These vaccines are two to three times more effective than the trivalent vaccine. Although the Americas have eliminated measles, Africa still suffers frequent outbreaks due to poor coverage. African ministers of health passed a resolution calling for measles elimination by 2020, at the 61st session of the WHO Regional Committee for Africa. The first key milestone to be reached before achieving measles elimination is to ensure that all countries in the region reach 90% coverage with the first dose of a measles-containing vaccine at national level. The conference noted that African countries need to improve their immunisation programmes if they are to save lives. Delegates resolved to enhance immunisation programme performance by strengthening human resources at all levels, from the grass roots to the national health department; timely collection and reporting of immunisation data; monitoring and evaluation of programme performance; adequate vaccine supply chain management; surveillance of adverse events following immunisation; and laboratory surveillance of vaccine-preventable diseases. It was strongly emphasised that it did not make good financial sense to introduce a new expensive vaccine into a dysfunctional immunisation system.

There was also much interest in the promotion of research and development and production of new vaccines and technologies on the continent. There was agreement that Africa needs to start manufacturing vaccines in order to drive prices down and ensure supply, as a matter of urgency. Cuba, a country which is poorer than many African countries and which has also faced major punitive sanctions, was lauded as an example for African vaccine manufacturers. Cuba is now self-sufficient in terms of vaccine production. African manufacturers were also called upon to take the initiative and manufacture the vaccines needed by Africans, for which there is a gap in the market, for diseases that are major problems in Africa and other low and middle-income countries. These include vaccines for TB, malaria, HIV, and neglected tropical diseases. Conference participants heard the latest developments in research on new TB, HIV, and dengue vaccines. The first announcement of the RTS,S malaria vaccine results in infants were presented. The vaccine was shown to be safe and although the vaccine efficacy of 30% is low; these results are encouraging in the quest for the first efficacious malaria vaccine. A strong emphasis was placed on (i) developing and promoting a vaccine advocacy action plan for Africa [8, 9] in the Decade of Vaccines, to encourage more allocation of resources for vaccine initiatives on the continent; and (ii) finding ways of ensuring sustainable financing of vaccines at country level [2]. It was also highlighted that appropriate measures should be taken to ensure that immunisation services meet the needs of recipient communities and that service providers should work with communities to ensure their involvement and participation. The delegates noted that there is power in joint action, as can be seen from the ability of the Pan American Health Organization ((http://www.paho.org/) to negotiate low prices for new vaccines and to eradicate/eliminate infectious diseases like polio, measles, and rubella. Delegates thus committed themselves, in the Cape Town Declaration on Vaccines, to continually advocate for political will and to hold legislators to account for ensuring access to vaccines and functioning immunisation services during this Decade of Vaccines [1]. The delegates committed themselves to advocate for sustainable financing for vaccines and immunisation programmes, including ring-fenced country budgets and innovative pooled pricing mechanisms to assist countries not eligible for GAVI support to access affordable vaccines. They would advocate for lower costs of immunisation programmes in Africa, perhaps through bulk buying and improved administration for rollout through the New Partnership for Africa′s Development (NEPAD). The delegates called for the integration of immunisation programmes into disease prevention programmes within a primary health care framework. They also recommended that immunisation programme performance be enhanced through strengthening human resource capacity at all levels; collection, collation and reporting of data; monitoring and evaluation; appropriate supply chain management; and strengthening of laboratories to provide quality surveillance for vaccine-preventable diseases. It was noted consistently throughout the conference that all efforts to improve vaccine access, delivery, and coverage will fail dismally without community involvement. Advocacy and communication efforts need to involve caregivers and patients. It is only by translating complex science into the language of those being vaccinated that the benefits and risks of immunisation will truly be understood. Delegates therefore, committed to effective communication and advocacy for immunisation at all levels of the health system as well as with traditional and faith leaders, civil society, and non-governmental organisations. Finally, the delegates confirmed their agreement with and commitment to the goals and strategic objectives of the GVAP and will hold African leaders responsible for its implementation. The conference was seen as useful by the attendees and their feedback in evaluating the conference was been overwhelmingly positive. The delegates recommended that the conference be held every two years.

## Conclusion

The first International African Vaccinology Conference, held in November 2012 in Cape Town, was the first international vaccinology conference in Africa, for Africa, and about Africa. The conference focused on critical vaccine and immunisation issues for Africa and the world. The delegates were unanimous that they cannot stand by while African children do not get a simple intervention that saves lives and reduces suffering on the huge scale that immunisation does. Universal immunisation coverage would allow millions of African children to lead normal healthy lives and develop into healthy adults, free from vaccine-preventable disability and disease.
